# Supplementary material for: An ultrastrongly coupled single terahertz meta-atom
Source: Nat Commun. 2022 May 9;13:2528. doi: 10.1038/s41467-022-29974-2 (PMC9085771; doi:10.1038/s41467-022-29974-2)
Supplement: Supplementary file 1 — Supplementary Information [file 41467_2022_29974_MOESM1_ESM.pdf]

An ultrastrongly coupled single terahertz meta-atom

Shima Rajabali[1,*], Sergej Markmann[1], Elsa Jöchl[1], Mattias Beck[1], Christian A. Lehner[2], Werner Wegscheider[2], Jérôme Faist[1], Giacomo Scalari[1,*]

[1]*Institute of Quantum Electronics, ETH Zürich, 8093 Zürich, Switzerland*

[2]*Laboratory for solid state physics, ETH Zürich, 8093 Zürich, Switzerland*

Optical images of the aSIL configuration, including the designed holder with proper clamps and metallic positioners for tightly holding the front and back lenses on the sample are displayed in Fig. S1.

Fig. S2 reports the Q-factor for the cSRR cold cavity measurement without the aSIL configuration as a function of the number of resonators in the Menlo Terasmart spectrometer. The red data points in panel (b) indicate the Q-factors extracted from the transmittance measurements in Fig. S2a. The blue data points and the blue fitted dashed line are related to the transmittance measurements of the cold cavity with the aSIL assembly which are shown and explained in the manuscript (Fig. 3). The resonant peak for the arrays with less than 9 resonators could not be resolved without the lenses.

The enhancement of the peak-to-peak value of the THz waveform in the time domain is reported in Fig. S3. The figure displays the transmitted signal in time domain through a single

cSRR on a GaAs substrate without (red curve) and with the aSIL configuration (black curve). The delay between the two signals corresponds to the optical thickness of the aSIL assembly. By employing the aSIL assembly on the single resonator sample, the peak-to-peak value of the signal in time domain is enhanced about 5 times.

Finite element simulation (by CST Microwave Studio software) shows the red shift of the resonant frequency for an infinite array of cSRRs in case of having a Si slab in front of the resonator (Fig. S4).

Fig. S5 represents a normalized transmittance through a single resonator (black curve) and a 60×60 array of cSRRs (orange curve) with the aSIL configuration in linear scale (the logarithmic scale is provided in the manuscript Fig. 3a). The difference between the resonant frequencies is 7 GHz.

Fig. S6 exhibits the extraction of the cyclotron decoherence rate for the GaAs 2DEG from the time domain measurements similar to the method explained in Ref[1]. The time traces are taken at a magnetic field value of $B = 2T$ from which the transmission field at $B = 0T$ is subtracted to deduce the magnetic field induced changes to the transmitted signal. A longer time trace in the cyclotron measurement with the lenses (Fig. S6b) compared to the one without the lenses (Fig. S6a) is due to the suppression of the echos from the interface between the back of the sample and the back lens which results in an increase of the frequency resolution. The extracted cyclotron decoherence rates are $\gamma_1 = \frac{1}{\pi\tau_1} = \frac{1}{\pi\times 8.65ps} = 36.8$ GHz and $\gamma_2 = \frac{1}{\pi\tau_2} = \frac{1}{\pi\times 14.74ps} = 21.6$ GHz for the measurements without and with aSIL assembly, respectively. The linewidth of the cyclotron

resonance for the measurement with the lenses is narrower (smaller γ).

To extract the mode dissipation or cavity decay rate (κ) and also calculate the Q-factor of the LP mode at its asymptotic limit, we first cut the signal in the time domain before the echo (from the cryostat windows). Then, the signal is digitally filtered by an ideal band-pass filter centered at the peak of the LP mode (bandwidth $\sim$ 400 GHz) to remove the effect of the second mode. In the end, similar to the extraction method for γ, the filtered signal is fitted with a sine function with exponentially-decaying amplitude ($y = y_0 + Aexp(t/\tau)sin(2\pi ft + \theta)$) to extract the oscillators' decay time (τ) and frequency (f) [1]. The normalized signal in the time domain from a single resonator (black curve) and a 60×60 array of the resonators (red curve) are reported in Fig. S7a. The filtered signal using an ideal bandpass filter (centered at LP mode with a bandwith of 400 GHz) and the fitted signal with a sine function with exponentially-decaying amplitude are provided in Fig. S7b. The Q-factors of the LP mode at B = 4T are then calculated as $Q_{single} = \frac{f_1}{Linewidth} = \frac{f_1}{1/\pi\tau_1} = \pi\tau_1 f_1 = \pi \times 16.3ps \times 301GHz = 15.4$ for the single resonator and $Q_{array} = \pi\tau_2 f_2 = \pi \times 7.87ps \times 280GHz = 6.9$ for the 60×60 array of resonators with the aSIL configuration.

Fig. S8 shows the accuracy of the fitted polariton branches using the Hopfield model for the transmittance measurements of the single resonator coupled to LL transitions of InSb QW exhibited in the manuscript (Fig. 5). The extracted maxima of the transmittance at each magnetic field (light blue and black circles) are fitted by blue (UP branch) and red (LP branch) solid lines in Fig. S8a and S8b. The normalized root mean square error (RMSE) of the fitting vs. the normalized coupling ratio are reported in Fig. S8c and S8d for the fittings in panel (a) and (b), respectively. The

best fit is where the normalized RMSE is minimized. More information about the fitting procedure can be found in the supplementary document of Ref[2].

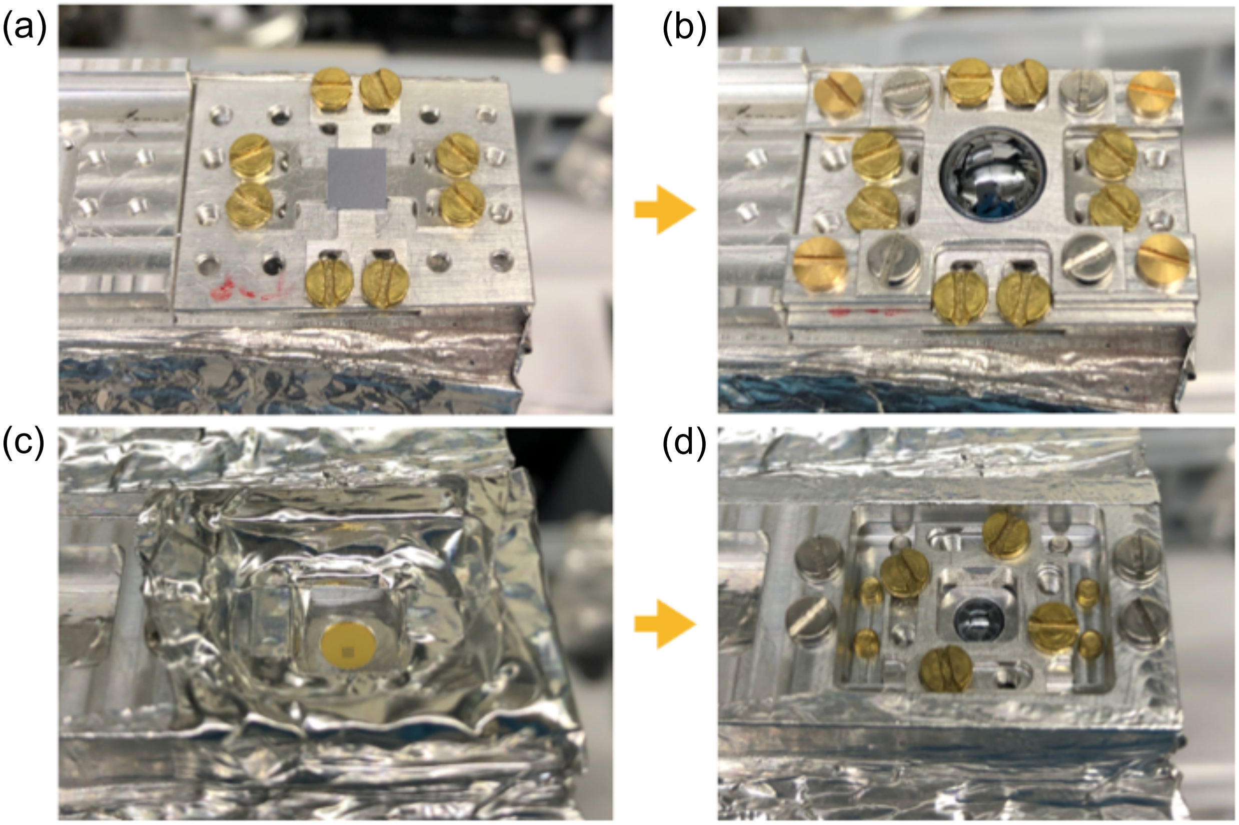

Figure S1: **Optical images of the aSIL configuration** (a), (b): Optical images of the back of the mounted sample without (a) and with (b) the back lens. (c), (d): Optical images of the front of the mounted sample without (c) and with (d) the front lens. The holder is wrapped with Aluminium foil, shown in panel (c), to block the transmission of air through the area without the sample.

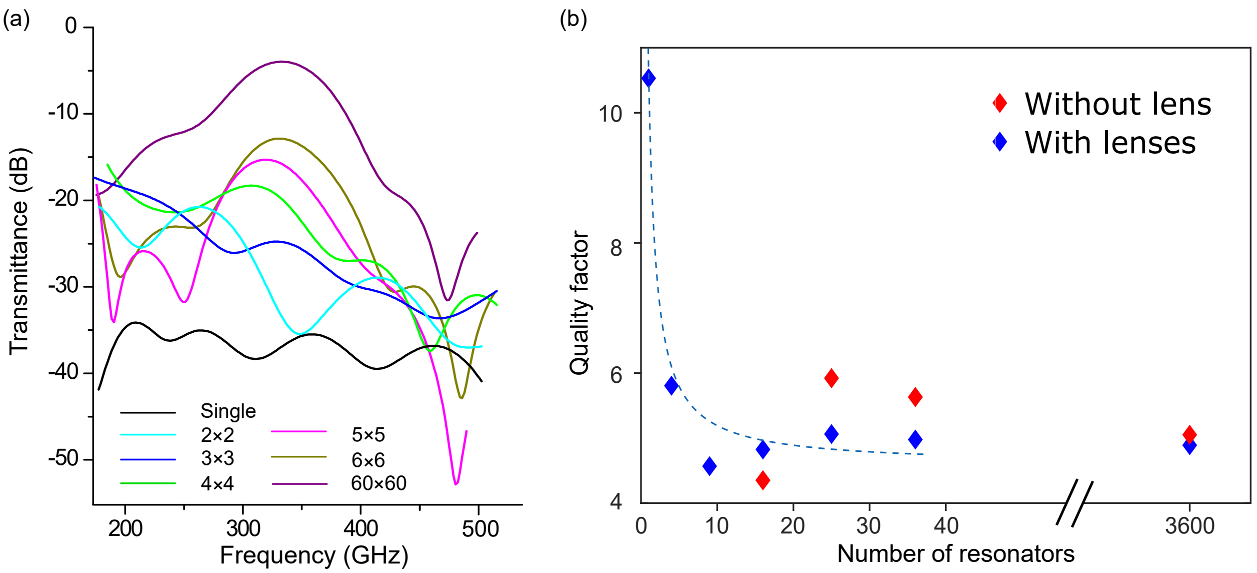

Figure S2: **The impact of reducing the number of resonators on resolving the resonant peak of cSRR arrays without using the aSIL configuration.** (a) Transmittance of $n \times n$ array of cSRRs ($n = 1$ to 6) and a large array with $n = 60$. These measurements are done without the aSIL assembly and the resonant peak for the arrays with less than 9 resonators could not be resolved. (b) is the Q-factor vs. the number of resonators extracted from the measurements without and with lenses. The red data points are calculated from the transmittance curves in panel (a) and the blue data points and the blue fitted dashed line are exactly the ones shown in the manuscript (Fig. 3b), related to the cold cavity measurements with the aSIL configuration.

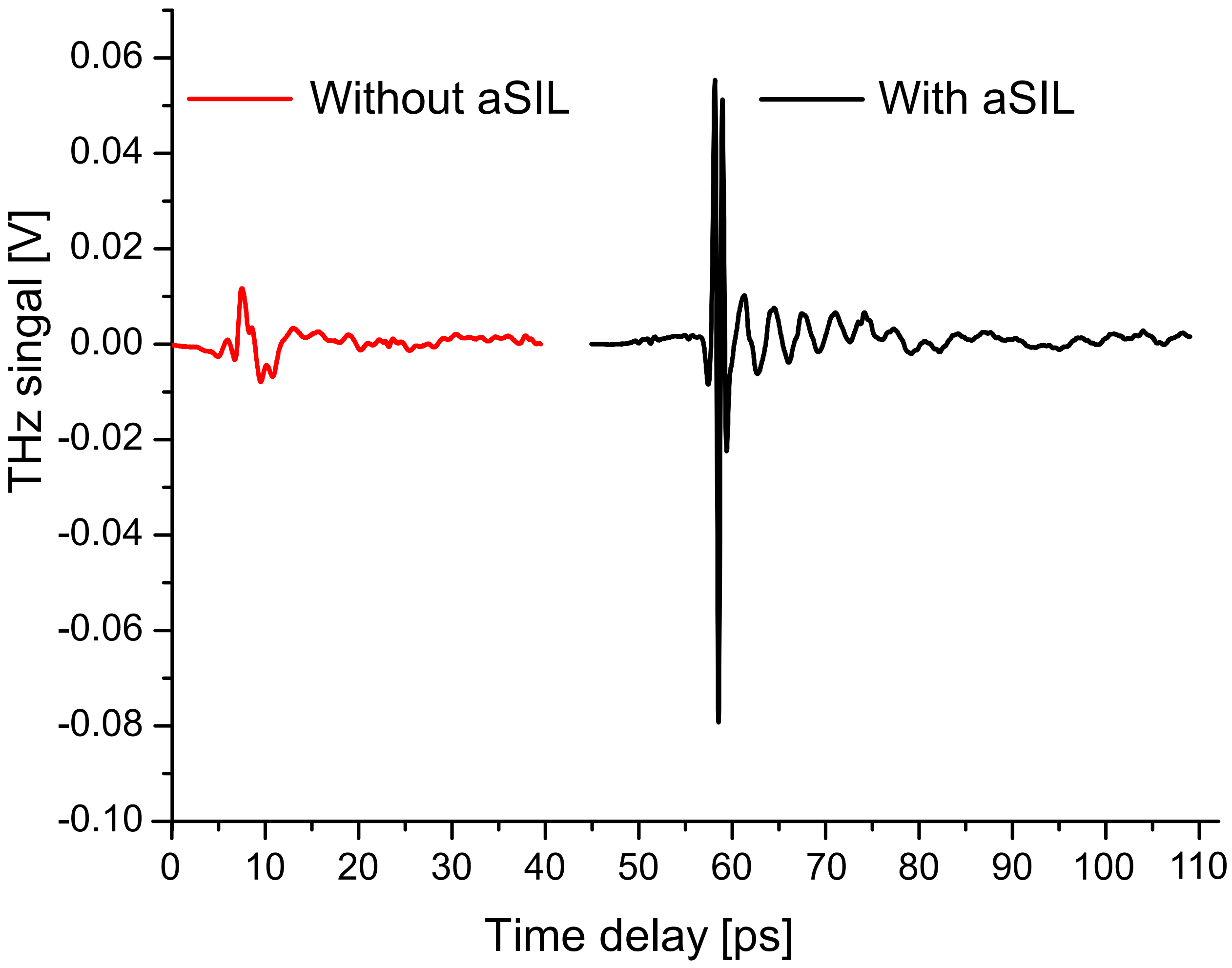

Figure S3: **The signal enhancement by employing the aSIL configuration** THz signal in time domain from a single resonator on GaAs substrate (cold cavity) without (red curve) and with (black curve) the aSIL configuration. The delay between the two signals corresponds to the optical thickness of the aSIL assembly.

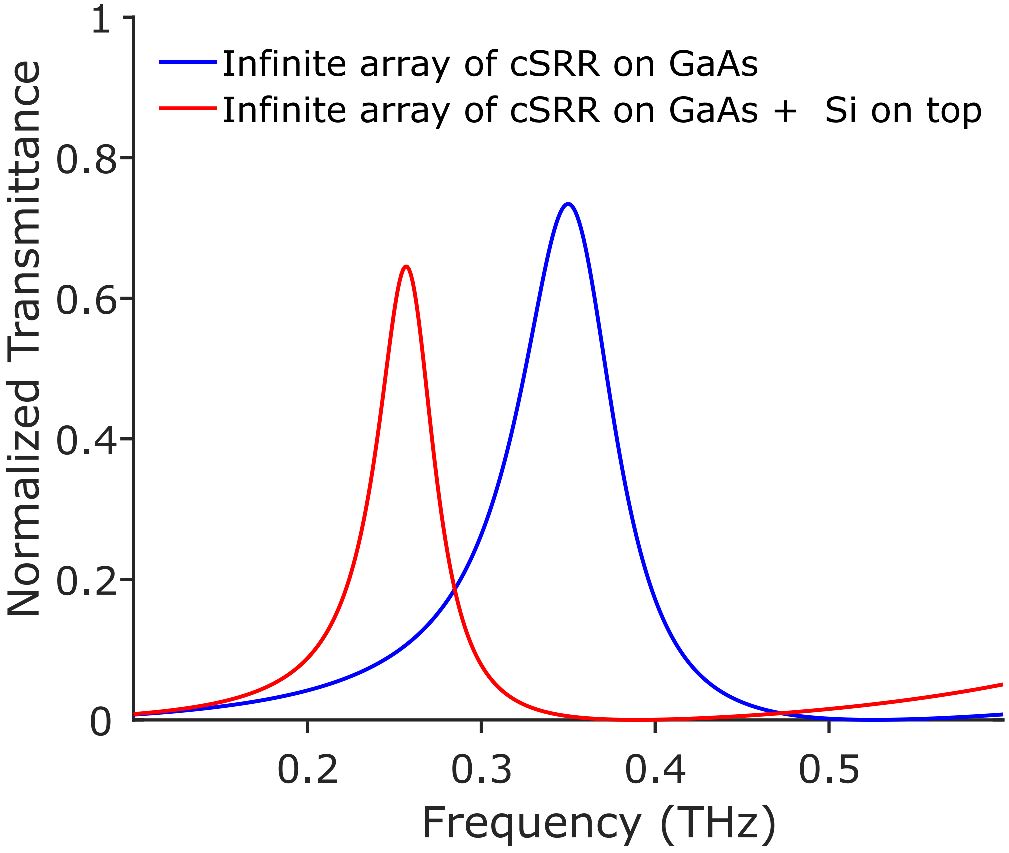

Figure S4: **Redshift of the resonant frequency due to presence of the Si in front of the resonators** The simulated normalized transmittance of an infinite array of cSRRs on a GaAs substrate without and with a Si slab in front of the resonators. The resonant frequency shifts from $350GHz$, in case of no Si slab in front, to $\sim 260GHz$ in case of having a Si Slab in front of the resonator plane.

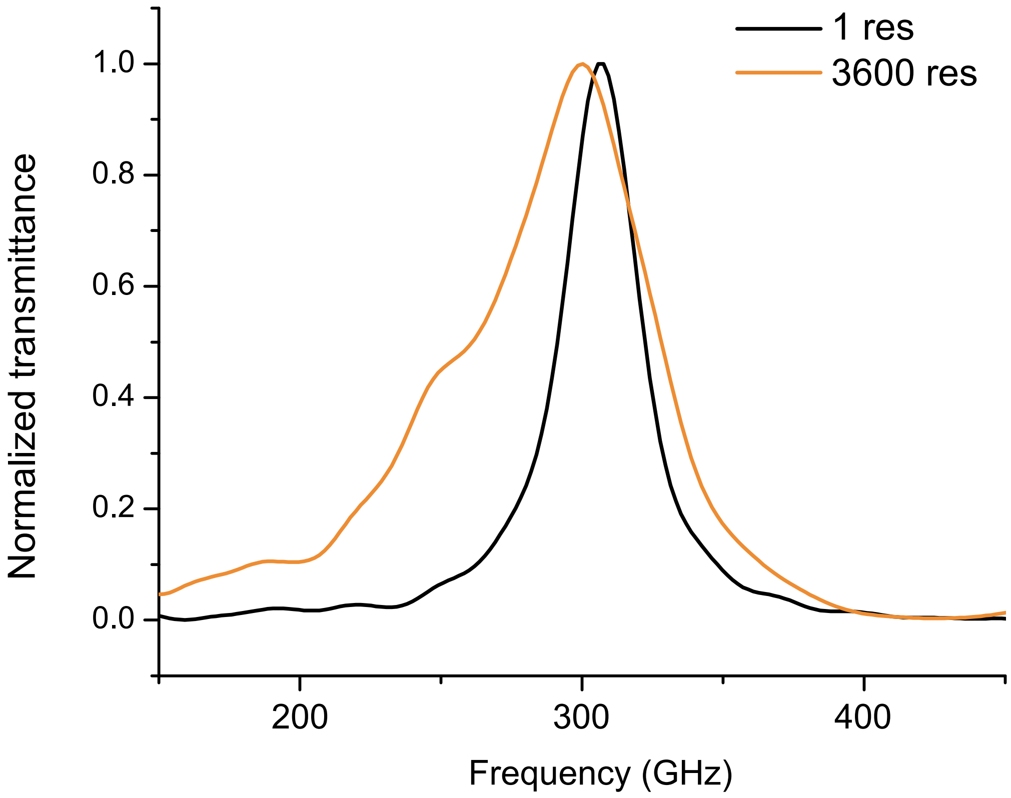

Figure S5: **A comparison between the cold cavity's resonant frequency of a single resonator and a large array of cSRRs in linear scale** The measurement of the cold cavity in Menlo (linear scale) for the single resonator and 60×60 array of resonators. Each curve is normalized to its peak value for a better comparison.

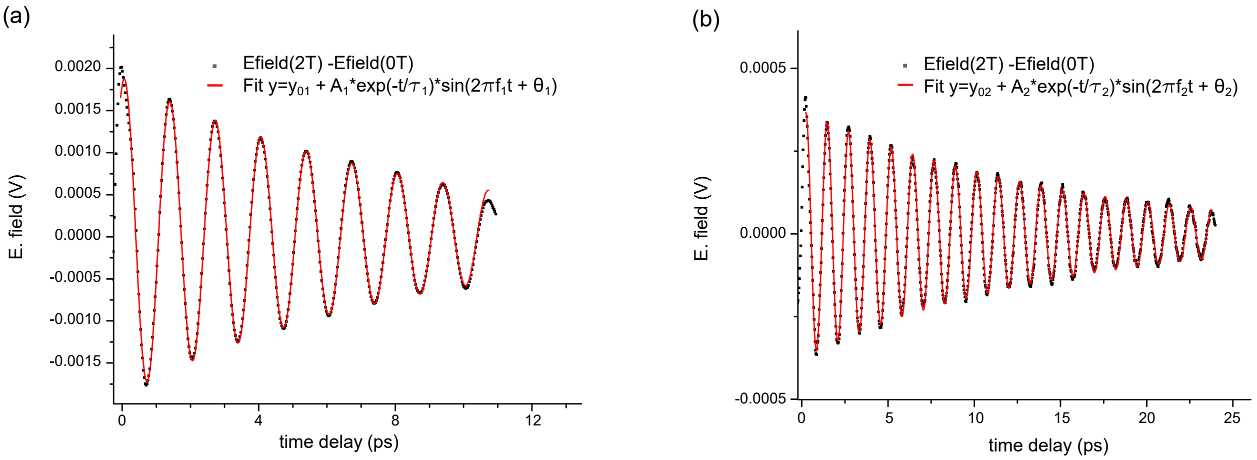

Figure S6: **Extraction of the cyclotron decoherence rate from the the direct analysis of time domain measurement** The time traces are THz-TDS for a GaAs 2DEG without (a) and with (b) the aSIL assembly at $B = 2T$. Similar to the method used in Ref[1], the time trace at $B = 0T$ is subtracted and the data (black dots) are fitted by sine functions with exponentially-decaying amplitudes to extract the cyclotron resonance decay times (τ_1, τ_2) and frequencies (f_1, f_2). The extracted cyclotron decoherence rates are $\gamma_1 = \frac{1}{\pi\tau_1} = \frac{1}{\pi\times 8.65ps} = 36.8GHz$ and $\gamma_2 = \frac{1}{\pi\tau_2} = \frac{1}{\pi\times 14.74ps} = 21.6GHz$ for the measurements without and with the aSIL configuration, respectively.

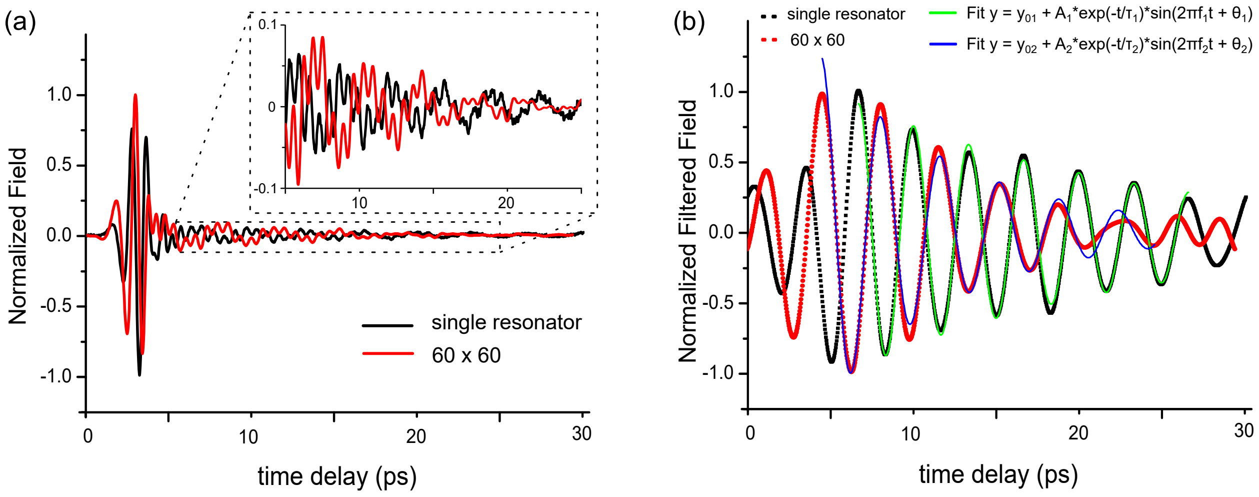

Figure S7: **Extraction of the cavity decay rate from the the direct analysis of time domain measurement** (a) Normalized THz signal in time domain from a single cSRR and a 60×60 array of resonators with the aSIL configuration. The inset shows the magnified image of the tail of the decayed signal to highlight the oscillations. (b) The waveforms in panel (a) are filtered with an ideal bandpass filter (centered at LP mode with a bandwith $\sim$ 400 GHz) and then fitted by sine functions with exponentially-decaying amplitudes to extract the oscillators' decay times (τ_1, τ_2) and frequencies (f_1, f_2). The extracted values for τ_1, f_1, τ_2, and f_2 are 16.3 ps, 301 GHz, 7.87 ps, and 280 GHz ,respectively.

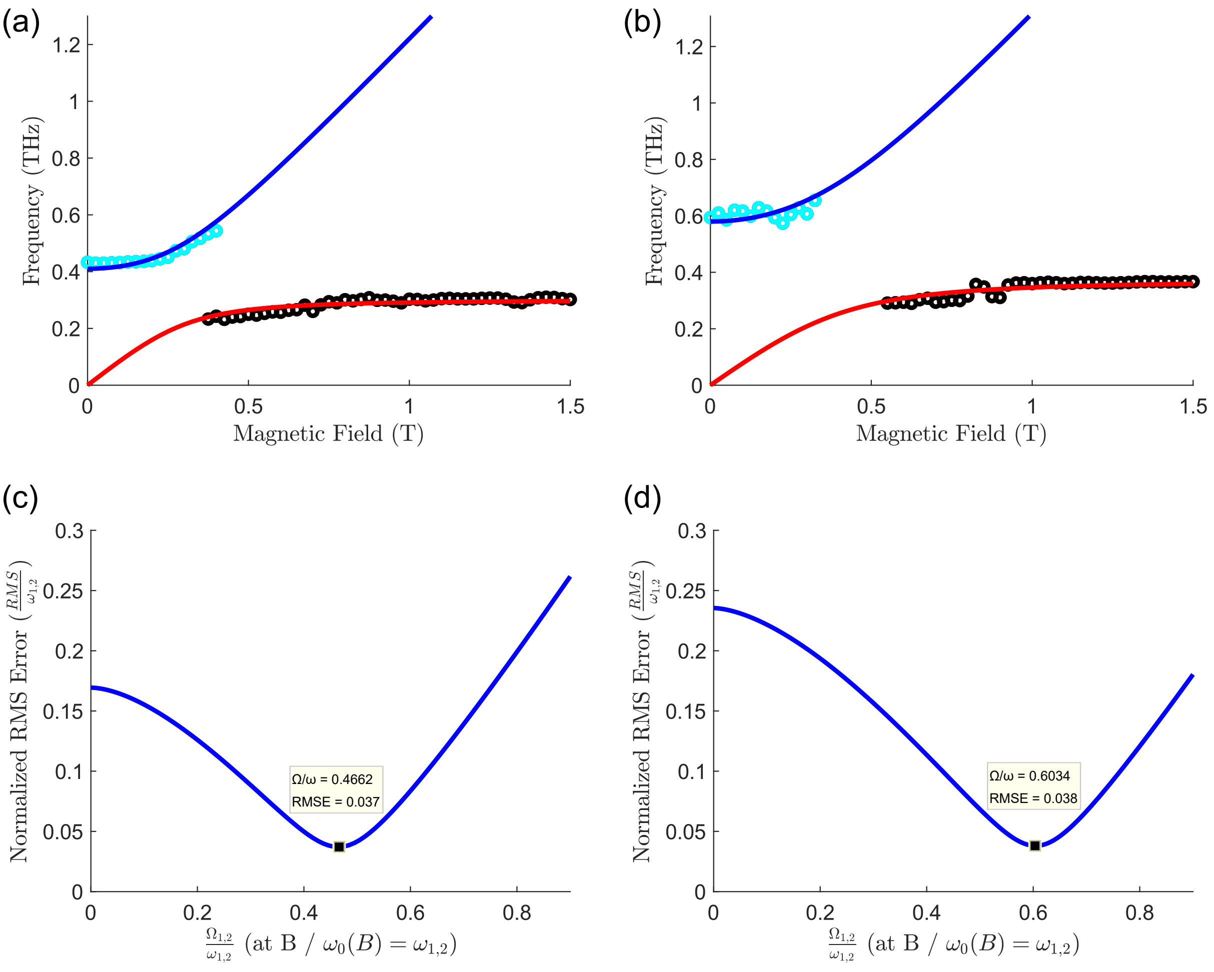

Figure S8: **The accuracy of fitting for the polariton branches of the coupled single resonator to LL transitions in InSb QW** (a) and (b): The extracted maxima of the transmittance measurement at different values of magnetic field (light blue circles for UP and black circles for LP) from the colormaps in Fig. 5 in the manuscript are fitted to the solid blue line (for UP) and solid red line (for LP) using Hopfield model. (c) and (d): The normalized RMSE of the fitting in panel (a) and (b), respectively, as a function of normalized coupling strength. The normalized coupling strength of the measurement is where the normalized RMSE is minimized[2].

1. Wang, X. *et al.* Direct measurement of cyclotron coherence times of high-mobility two-dimensional electron gases. *Opt. Express* **18**, 12354–12361 (2010).

2. Scalari, G. *et al.* Ultrastrong coupling of the cyclotron transition of a 2D electron gas to a THz metamaterial. *Science* **335**, 1323–1326 (2012).
